# Rapid Sensing: Hand-Held and Portable FTIR Applications for On-Site Food Quality Control from Farm to Fork

**DOI:** 10.3390/molecules28093727

**Published:** 2023-04-26

**Authors:** Nur Cebi, Hatice Bekiroglu, Azime Erarslan, Luis Rodriguez-Saona

**Affiliations:** 1 Food Engineering Department, Chemical-Metallurgical Faculty, Yıldız Technical University, 34210 Istanbul, Turkey; 2 Bioengineering Department, Chemical-Metallurgical Faculty, Yıldız Technical University, 34210 Istanbul, Turkey; 3 Department of Food Science and Technology, The Ohio State University, 100 Parker Food Science and Technology 2015 Fyffe Road, Columbus, OH 43210, USA

**Keywords:** hand-held FTIR, portable FTIR, food quality, food authenticity, food adulteration, food integrity, chemometrics

## Abstract

Today, one of the world’s biggest problems is the assurance of food integrity from farm to fork. Economically motivated food adulteration and food authenticity problems are increasing daily with considerable health and economic effects. Early detection and prevention of food integrity-related problems could be provided by the application of effective on-site food analysis technologies. FTIR spectroscopy coupled with chemometrics can be used for the rapid quality control of a wide variety of food products with fast, high-throughput, accurate and nondestructive analysis advantages. In particular, hand-held and portable FTIR instruments have the potential to surveil food quality and food safety in various critical segments of the food supply chain. In this review, we explore the abilities of hand-held and portable FTIR spectrometers combined with multivariate statistics to conduct a quality evaluation of various food products in terms of food adulteration and authenticity issues. An examination of the literature showed that comparable results were obtained based on detection limits, correlation coefficient (R^2^) values, standard error values and discrimination power by using both portable/hand-held FTIR spectrometers and benchtop FTIR spectrometers. In conclusion, this review highlights the potential usefulness of portable and hand-held FTIR spectrometers combined with chemometrics for maintaining the food quality through the presentation of various applications that may shed light for on-site food control at any point of the food supply chain.

## 1. Introduction

Today, food adulteration and contamination problems are increasing at an alarming rate across the world. In this context, the concept of food integrity is becoming more important each day. The term “integrity” comprehensively describes the characteristic features of food products in terms of their nutrition, health, taste, safety, authenticity, traceability, ethics and sustainability aspects [1]. In general, the food supply chain has a complex structure including the provider, producer, distributor, retailer and consumer who are involved in the farm to fork process [2]. The complexity and diversity of the food supply chain make the system vulnerable to a wide variety of food fraud and food safety problems. Previous studies reported that global food fraud costs between $10 billion and $15 billion per year according to grocery manufacturers’ estimations [3]. Food fraud and food adulteration problems mostly occur through economic motivations with the purpose of economic gains and may result in food quality and/or food safety problems [4]. The types of food fraud can be summarized as replacement (1), addition (2) and removal (3) with possible risks to health, the economy, food quality and food safety [4]. A major concern of food fraud is the threat to public health. This was exemplified by the melamine milk crisis, which showed that melamine was deliberately added to milk in China (2008) to increase the apparent protein content of the milk [5]. A considerable number of incidents of food fraud have been reported, and the leading food categories can be listed as olive oil, fish and seafood, milk and milk-based products, honey, maple syrup and other sweeteners, fruit juice, coffee and tea, spices and organic foods and products [4]. Consumer perceptions have changed as a result of the food fraud cases being acknowledged by the public. Consumers are demanding more information about foods because of their doubts and suspicions about the food supply chain.

**Figure 1:**
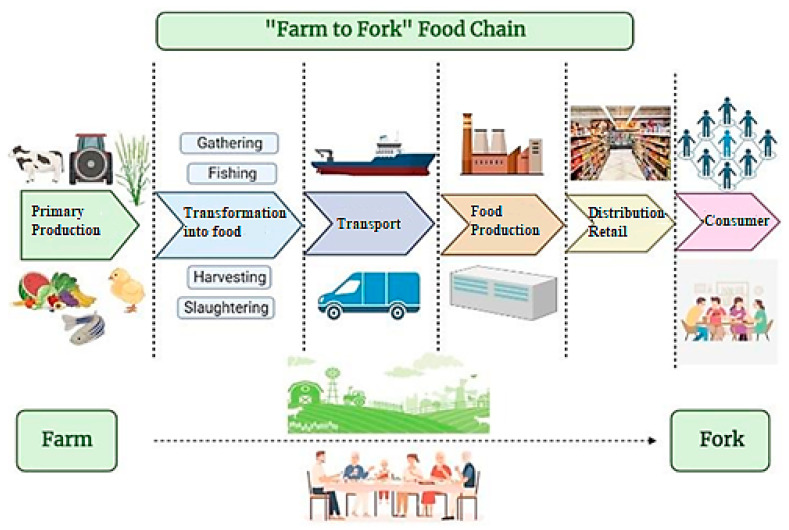
An illustration of the “Farm to Fork” food chain from primary production to the consumer.

As a result, there is a need for new and effective control systems built by responsible authorities to monitor food safety, food authenticity and food sustainability in terms of food integrity. There have been various efforts to identify food adulteration and food authenticity by using laboratory-compatible equipment; however, the on-site applicability of the developed methodologies in the field is questionable. Hand-held and portable instruments have groundbreaking potential to maintain food quality and food safety in various critical segments of the food supply chain from farm to fork. An illustration of the “Farm to Fork” food chain from primary production to the consumer is presented in Figure 1.

FTIR spectroscopy is a popular monitoring technique used by both the sector and regulatory control laboratories for the determination of food adulteration and food authenticity issues [6]. A number of research articles have been dedicated to the development of methodologies for food fraud and food-integrity-related issues by using FTIR spectroscopy in conjunction with multivariate statistics. This review summarizes the relevant work done so far on the feasibility of using portable, hand-held FTIR equipment combined with chemometrics to provide on-site food control from farm to fork.

## 2. Fourier Transform Infrared Spectroscopy

The heart of the FTIR (Fourier transform infrared spectroscopy) spectrometer is the interferometer system, which was developed by Albert Abraham Michelson, who received the Nobel Prize in 1907 for his optical precision instruments and the spectroscopic and metrological investigations carried out with their aid. Fourier transform infrared (FTIR) spectroscopy allows fast, high-throughput, accurate and nondestructive analysis by operating in the mid-infrared region of the electromagnetic spectrum.

FTIR spectroscopy can be efficiently employed in a wide variety of disciplines, such as food, pharmaceuticals, cosmetics, chemicals, criminalistics, forensic science and more, since functional groups of the covalently bonded molecules have shown IR absorption. Furthermore, IR spectroscopy has been reported to be among the “Category A” techniques with “maximum potential discrimination power” by the Scientific Working Group for the Analysis of Seized Drugs (SWGDRUG) [7]. Additionally, mid-infrared (MIR) spectroscopy has the ability to provide data about structure–functionality relationships in complex food matrices. In this context, MIR spectroscopy has a high ability to be used for the quality evaluation of food and agricultural products [8].

**Figure 2:**
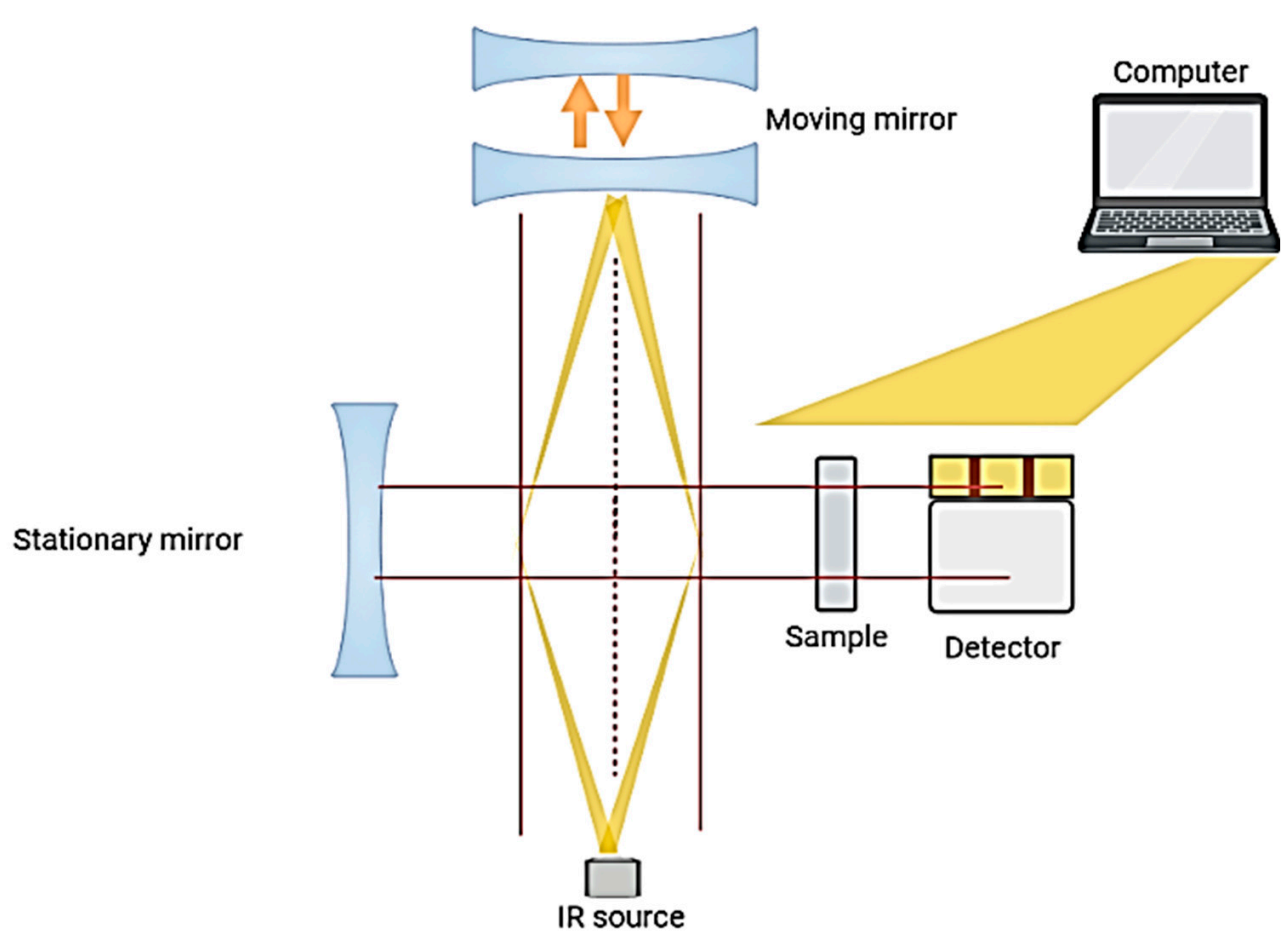
Schematic illustrations of the components and basic sections of the FTIR spectrometer.

Infrared spectroscopy studies the interaction between matter and IR radiation. The effect of IR radiation is measured through the absorption of IR frequencies by the sample which is located in the pathway of the IR beam [9]. As the main principle, the functional group of the analyte absorbs the infrared light at a specific wavenumber (cm^−1^), and this specific wave number can be assigned to the molecule structures [10]. Recently developed FTIR systems are composed of three main sections: a radiation source, an interferometer and a detector. Figure 2 shows the main working scheme, including the IR source, detector, Michelson interferometer, beam splitter, fixed mirror and moving mirror of the FTIR system. The interferometer is one of the most important sections of modern FTIR systems, since it regulates the infrared beam by splitting it into two paths towards fixed and movable mirrors. The interference pattern is formed by the combination of the reflected beams. Repetitive interference signals are produced and measured as a function of the optical path difference by a detector. An interferogram is the signal format of the FTIR spectrometer that contains all frequencies that constitute the infrared spectrum. However, it has a very complex structure because it contains many wavelengths. Thus, a mathematical process known as the Fourier Transform is applied to the interferogram to obtain the spectrum, which shows the relationship between the intensity (absorbance or transmittance) and frequency (wavelength) [11]. The IR spectrum of a molecule shows fingerprinting spectral properties and represents the chemical structure through absorption bands of the molecular functional groups in the spectral range of 4000–400 cm^−1^ [9]. In other words, a typical FTIR spectrum can be defined as a chemical fingerprint, since it reflects the unique spectral properties that are built by the vibration of functional group bonds, such as stretching vibrations and bending vibrations [12]. FTIR spectra can be used effectively for the identification of food adulteration, food quality and authenticity issues, since they reflect the overall chemical composition of the investigated materials. On the basis of the powerful aspects of IR spectroscopy, this technique can be used for the identification, characterization and quantification of selected analytes. Consequently, one can conclude that the FTIR technique can be employed for the at/in/online quality evaluation of food and agricultural products. Furthermore, previous studies reported that FTIR spectroscopy provides rich information about materials, and it can be defined as being perhaps more sensitive for the detection of minor compounds in complex food matrices when compared with NIR (near-infrared) technology [13]. 

## 3. Preprocessing and Chemometrics

According to previous reports, fingerprinting analysis has been accepted as a methodology by the World Health Organization (WHO) for the quality and safety evaluation of herbal products [14]. In particular, the combination of fingerprinting techniques with multivariate statistical analysis can ensure a comprehensive assessment of the chemical compositions of the investigated data set by revealing the intrinsic relationships among elements [15].

Prior to the exact chemometric studies, the application of pretreatment techniques to spectral data may reduce and eliminate variation that has arisen from the experimental and methodological conditions. The most commonly used techniques for the pretreatment of vibrational spectroscopy data are baseline correction, smoothing, mean centering, derivatization (first or second), normalization and multiplicative scatter correction [9,14]. Briefly, baseline correction is performed to obtain a flatten baseline and reduce the variety arising from the alterations in the baselines of the data set. As a smoothing technique, Savitzky–Golay smoothing is widely used for the elimination of the noise and to increase the spectral information [16]. Previous contributions reported that second derivatization was applied to increase the number of distinguishing spectral properties to obtain a better spectral resolution [9]. The data obtained by sensitive equipment, such as the FTIR spectrometer, are prone to the effects of the environmental and experimental conditions. In this case, normalization-preprocessing can be used effectively to decrease the spectral differences in the heterogeneous data [17].

The application of vibrational spectroscopy techniques accelerated the collection of large spectral data sets also known as “big data” [18]. Chemometrics can be used efficiently to extract distinctive information and release the hidden relationships among then evaluated variables [19]. Multivariate analysis methods can be divided into two major groups based on their quantitative and qualitative abilities. Quantitative methods can be listed as nonlinear methods, linear methods, artificial neural networks (ANNs), nonlinear PLS, multiple linear regression (MLR), principal component regression (PCR), partial least squares (PLS) and least squares support vector machines (LS-SVM). Qualitative methods can be listed as unsupervised methods, supervised methods, cluster analysis (CA), ANNs, principal component analysis (PCA), Fisher discriminant analysis (FDA), *k*-nearest neighbor (KNN), linear discriminant analysis (LDA) and soft independent modeling of class analogy (SIMCA) [20]. Previous reports classified the unsupervised techniques into PCA, cluster analysis, factor analysis, similarity analysis and hierarchical cluster analysis (HCA). Classification techniques are beneficial for displaying the classification pattern of the elements in the form of various graphical illustrations. Cluster analysis (CA) techniques mainly group the investigated elements into clusters based on their similarities and dissimilarities by using different clusterization algorithms, such as linkage clustering, Ward’s algorithm and the centroid method. In supervised techniques, collected spectra from the data set are introduced as known groups in the training stage to develop a function or build (train) a mathematical model. Soft independent modeling of the class analogy, linear discriminant analysis and canonical variate analyses can be used to classify samples based on spectral information. Additionally, artificial neural networks (ANNs) are supervised mathematical techniques that can be used to build data patterns. ANNs are capable of predicting categorical and quantitative variables [21].

## 4. Portable FTIR Systems

Recently, FTIR spectrometers have not been restricted to laboratory-bench-compatible instrumentations. Portable and hand-held forms of the FTIR spectrometers can be utilized when real-time data acquisition is needed in the field. In other words, miniature versions of vibrational spectroscopy equipment have opened a new door into the food industry for on-site and real-time evaluation of food quality and food production processes. Portable FTIR instruments are built by the combination of miniaturized interferometers and circuits in the structure of the system [22]. According to previous contributions, portable and hand-held MIR spectrometers may have some limitations related to the required system components, such as moving parts and quantum-type pyroelectric detectors, since these components require cooling to decrease the thermal noise level [23]. However, previous contributions stated that MIR spectroscopy has advantages when compared with NIRS (near-infrared spectroscopy), since MIRS provides unique information about the functional groups of organic materials, and it is possible to quantify specific compounds based on their absorption intensities [23].

**Figure 3:**
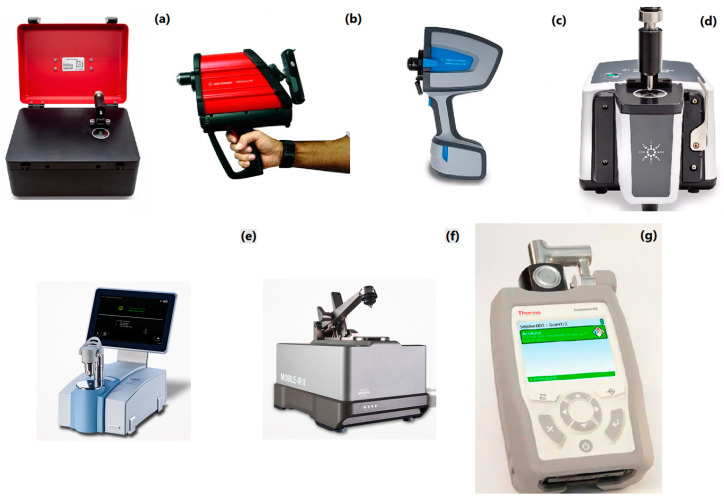
Pictures of some commercial portable and hand-held FTIR spectrometers. Agilent 4500 FTIR (**a**); Agilent 4100 Exoscan FTIR (**b**); Agilent 4300 Hand-held Portable FTIR (**c**); Agilent Cary 630 FTIR (**d**); Bruker Alpha II Compact FTIR (**e**); Bruker Mobile-IR II (**f**); Thermo Scientific TruDefender FTX (**g**) [25,26,27,28,29,30,31].

It was reported that a limited number of hand-held FTIR spectrometers that operate with the Michelson interferometer are traded by commercial brands. There have been efforts to miniaturize FTIR systems: by employing gratings composed of pyroelectric lead zirconate titanate (PZT) instead of the Michelson interferometer and by adding improvements through Fabry–Pe’rot interferometry combined with a pyroelectric sensor. Although these advances have provided simplicity and lower costs, they have led to higher spectral noise and a lower resolution [24]. According to our exploration, there is a limited variety of hand-held and portable FTIR systems, and some of these products can be listed as the Agilent 4300 hand-held portable FTIR, Agilent Cary 630, Agilent 4100 Exoscan, Agilent 4500, Arcoptix FTIR-rocket, Bruker Alpha II, Bruker Mobile IR II and the ThermoFisher Scientific TruDefender. Pictures of some commercial portable and hand-held spectrometers are presented in Figure 3.

## 5. Implementation of Portable and Hand-Held FTIR Spectroscopy to Food Control

Globally, the food supply chain, from farm to fork, consists of various stakeholders. The complexity and variety of the supply chain lead to vulnerability and concerns about food quality and safety [6]. The current situation indicates that stakeholders and authorized institutions should take the necessary actions regarding these issues and increase their activities associated with food-control applications.

FTIR spectroscopy has been employed as an advantageous spectroscopy technique in food matrices with appealing properties such as fast scanning, low-cost operation, high-throughput analysis, green-compatible measurement and unique chemical fingerprinting. To date, the combination of FTIR spectroscopy with multivariate statistical methods has been effectively used for the determination of food authenticity and food adulteration problems in a wide variety of food products, such as herbal products, fruits, agricultural products, oils, dairy products and more [32]. In particular, portable and hand-held vibrational spectroscopy techniques, such as FTIR spectroscopy, have high potential for “on-site” and “in the field” quality evaluations of agricultural and food products in different segments of the food supply chain without an instant need for any elaborate and high-cost analysis. Various studies have evaluated the ability of portable spectrometers to be used for quality evaluations of foods [13,23,24]. In previous reports, portable and hand-held FTIR applications were presented as a subsection in a limited amount of literature. Differently from these contributions, our review article mainly focuses on FTIR spectroscopy, portable and hand-held FTIR spectroscopy and food control applications. Additionally, to the best of our knowledge, we have presented all research articles produced to date about the use of portable and hand-held FTIR applications combined with chemometrics for the quality control of food products. These studies included a wide variety of food products, such as fats, oils, vegetables, milk, dairy products, meats, cereals, nuts and other products.

Table 1 presents the previous studies performed by using portable and hand-held FTIR spectrometers for determination of the authenticity, quality, safety and other essential parameters in foods. Fats and oils are important food ingredients and food products in terms of food quality properties and food authenticity. The literature shows that most of the studies performed so far have included oils, fats and fat-containing food products. To the best of our knowledge, one of the first studies was performed by Birkel and Rodriguez-Saona [33] who evaluated the performance of a hand-held FTIR sensor combined with PLSR chemometrics for quantification of the trans fat content. Additionally, a benchtop FTIR was employed as a reference methodology. On the basis of the used methodologies, hand-held FTIR was successful to predict the trans fat content of edible oils, such as peanut, safflower, corn and coconut. Their results showed that it is possible to obtain similar results (R^2^ > 0.98) by using both benchtop and hand-held FTIR equipment On the basis of the requirements of trans fat labeling in the US and other countries, Mossoba et al. [34] evaluated the ability of a portable FTIR spectrometer equipped with a nine-reflection diamond ATR crystal for the determination of the total trans fatty acid concentration. They showed that it was possible to obtain an enhanced sensitivity by using 9-bounce portable ATR-FTIR equipment at the detection limit of around 0.34% trans fats (percentage of total fat). It is known that label verification and label control are highly important concepts for the control and maintenance of food quality. Additionally, the allowed trans fat limits may vary according to the regulations of each country. Their results showed that the portable 9-bounce ATR-FTIR can be used effectively and rapidly in the field for the detection of the trans fat content of various food products and dietary supplements [34]. Additionally, Mosabba et al. [35] evaluated the performance of a novel portable FTIR operating in transmission mode for rapid detection of the total trans fatty acid content of different fast products, such as hamburgers, chicken tenders, French fries and apple pies. They reported that a similar detection value limit of around 0.58% for trans fatty acid methyl esters was obtained by using both the portable-transmission-mode FTIR spectrometer and the benchtop ATR-FTIR spectrometer. Another study by Mosabba et al. [36], which supports the high capability of portable FTIR equipment, showed that comparable results for the trans fatty acid methyl ester (FAME) content of fat samples extracted from fast-food samples were obtained by using a benchtop FTIR and portable FTIR equipment.

In another study, Allendorf et al. [37] evaluated the performance of a hand-held mid-infrared spectrometer combined with chemometrics to monitor the oxidation process and determine the fatty acid composition of frying oils. PLSR models that predict the peroxide value (meq/kg) and free fatty acid content (%) showed that favorable R^2^ (>0.959) values were obtained, and SECV values were comparable for both benchtop and hand-held FTIR equipment. Additionally, the hand-held ATR-MIR system provided reasonably favorable R^2^ values of ≥98 for the determination of saturated, monounsaturated and polyunsaturated fatty acid contents (%) by using the PLSR statistical analysis. These results show the ability of portable and hand-held FTIR systems to monitor the oil quality simultaneously, so they could be employed in the edible oil industry at any point of the food supply chain for online and in situ evaluations.

**Figure 4:**
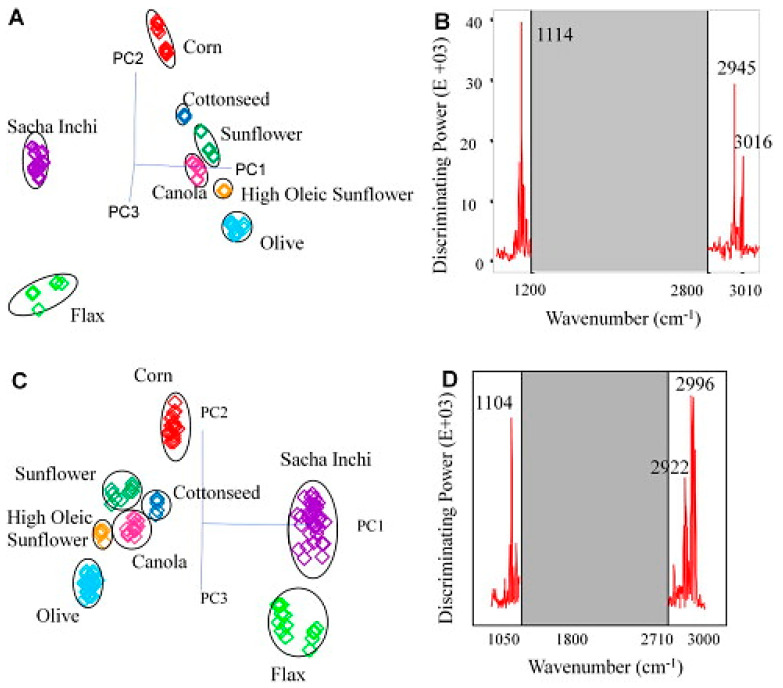
Three-dimensional PCA plot obtained from the SIMCA analysis of the spectral data acquired by the benchtop mid-IR ATR spectrometer (**A**); 3D-PCA plot obtained from the SIMCA analysis of the spectral data acquired by the hand-held portable mid-IR ATR spectrometer (**C**); SIMCA discrimination plots for the benchtop spectrometer (**B**); SIMCA discrimination plots for the hand-held portable spectrometer (**D**). Reproduced with permission [38]. Copyright 2012, Elsevier.

In a different study, Maurer et al. [38] utilized a temperature-controlled ZnSe ATR mid-infrared benchtop and a mid-infrared portable, hand-held spectrometer (TruDefender™, Thermo Scientific) combined with SIMCA chemometrics for the characterization and authentication of omega-3 rich sacha inchi (*Plukenetia vobulis* L.) oil. The authors used FTIR techniques combined with PLSR chemometrics to track the peroxide value (PV) and free fatty acid (FFA) contents. The correlation coefficient (R^2^) values of the PLSR models were determined to be higher than 0.9 for both the benchtop and portable FTIR spectrometers. The SIMCA pattern recognition results obtained by Maurer et al. [38] are presented in Figure 4. The SIMCA 3D projection plots of the data set obtained by the ATR-MIR benchtop spectrometer and the portable, hand-held FTIR system are illustrated in Figure 4A and Figure 4C, respectively. The oil species were grouped in relation to their compositional similarities and differences based on the principal component analysis. It can be seen that well-separated clusters were obtained, and sacha inchi oil was successfully discriminated from other species, such as corn, cottonseed, sunflower, canola, high oleic sunflower, olive and flax oils by using both the benchtop and portable FTIR spectrometers. Briefly, their results show that the ATR-MIR hand-held spectrometer showed a similar ability to carry out an authenticity evaluation when compared to benchtop ATR-MIR equipment. Notably, the authors reported that slightly more noise was observed in the FTIR spectra obtained by the portable, hand-held spectrometer.

Another study evaluated the ability of the portable ATR-FTIR spectroscopy technique combined with the PLSR analysis to rapidly determine the protein content of microencapsulated fish oil powders. The portable ATR-FTIR predicted a total protein content that was successfully correlated with the reference HPLC values with an R^2^ value of 0.975 [39]. The chromatographic reference method is laborious when compared with the rapid and robust FTIR technique. The methodology they developed could be used as an alternative technique for the online and real-time evaluation of microencapsulated fish oil powders for quality determination in the supply chain.

Successful quantification of the major and minor fatty acids in marine oil omega-3 dietary supplements was performed by employing a portable FTIR spectrometer combined with PLSR methodologies. Reasonably favorable R^2^ values > 0.95 were obtained for all models, and the predictive quality of the models were classified as excellent (*n* = 3), very good (*n* = 4), good (*n* = 1) and fair (*n* = 1) [40]. The study by Karunathilaka et al. [40] showed that portable FTIR can be used as alternative equipment for the determination of the fatty acid contents of marine oil supplements in the field, instead of using benchtop spectrometers and chromatography systems, to maintain the product quality and to control product properties on the basis of regulatory concerns. Another study by Karunathilaka et al. [41] employed both benchtop and portable FTIR systems combined with PLSR chemometrics for the accurate quantification of trans fat at low concentrations (<1% of total fatty acids) in edible oils and fast-food lipid extracts. According to their results, it was possible to build robust PLSR calibration models based on high R^2^ values and low root mean square error of cross-validation (RMSECV) values for a wide variety of samples, including edible oils, lipid extracts from hamburgers, chicken tenders/nuggets, French fries and apple pies by using both benchtop FTIR and portable FTIR systems.

Additionally, Karunathilaka et al. [42] utilized portable FTIR equipment in combination with PLSR for the rapid quantification of EPA (eicosapentaenoic acid), DHA (docosahexaenoic acid) and other fatty acids in marine oil omega-3 supplements. According to the model validation results, the FTIR-PLSR models showed excellent predictive capabilities for the quantification of EPA and DHA contents.

Salas-Valerio et al. [43] performed a comprehensive study on the determination of trans fat levels in commercial butter and margarine in the Peruvian market. The authors used a portable (five reflections) FTIR spectrometer, hand-held (single-reflection) FTIR spectrometer and a palm-sized near-infrared (FT-NIR) sensor. According to their report, the best quantitative analysis model was obtained by using a five reflections FTIR-ATR system, followed by single-reflection FTIR-ATR and FT-NIR systems. Additionally, they stated that portable and hand-held systems showed sufficient efficiency in the prediction of the contents of trans fat and other major fatty acids in margarine and butter, since these systems provide the unique fingerprint properties of molecules, allowing them to distinguish the compositional diversity.

Besides fats, oils and lipids, several studies have evaluated the authenticity and quality of various vegetables, such as tomatoes, potatoes and onions, by using portable and hand-held FTIR systems. For example, Wilkerson et al. [44] evaluated the potential of benchtop and portable FTIR systems combined with PLSR to be used for the quality determination of processed tomatoes on the basis of the standard error of cross-validation (SECV) and correlation coefficient of cross-validation (R^2^) values. They obtained statistically reliable results, and the authors demonstrated the high potential of the IR portable unit to be used for the high-throughput quantification of quality parameters in tomatoes in the field. Another study used a portable infrared instrument for the rapid detection of sugar, asparagine and glutamine levels in raw potato tubers, and the authors reported that the application of a portable FTIR system combined with chemometrics resulted in robust models with low standard error of prediction (SEP) values and high residual predictive deviation (RPD) values [45]. The study by Ayvaz et al. [46] showed the strong ability of a portable infrared system paired with PLSR to quantify the chlorogenic acid, total phenolic, total monomeric anthocyanin, sucrose, glucose, fructose and reducing sugar contents of potato samples. 

The application of rapid methodologies in the field by using portable systems may provide benefits for crop management and production systems. Krahmer and co-authors utilized a portable ATR-FTIR spectrometer to profile nonstructural carbohydrates in onion (*Allium cepa* L.). They classified onion in relation to parameters such as fresh market, storage and dehydrator types by using the hierarchical cluster analysis (HCA) and found that the clusterization pattern was coherent with the nonstructural carbohydrate patterns obtained by a high-pressure liquid chromatography–evaporative light scattering detection (HPLC-ELSD) analysis [47]. Another valuable study used a portable ATR-FTIR spectroscopy instrument to build a rapid and robust methodology for nitrate monitoring during the plant growth process in leafy vegetables, such as cabbage, spinach, celery and lettuce. The results showed that portable ATR-FTIR spectroscopy combined with the Euclidean distance-modified extreme learning machine (ED-ELM) model is a promising methodology for the determination of the nitrate content in vegetables in terms of assessing the food and agriculture quality [48].

The dairy industry has considerable importance in terms of the nutrition concerns of the public, ranging from infants to the elderly community. In this context, the quality determination of dairy products comes into prominence for the maintenance of the safety and reliability of dairy products, such as milk and milk powder. Limm et al. [49] employed portable ATR-FTIR equipment in conjunction with SIMCA chemometrics for the determination of melamine in milk powder. Their results showed that the portable ATR-FTIR system combined with SIMCA methodology has a strong ability to enlighten economically motivated adulteration issues, such as the melamine scandal.

Another important food category is composed of meat and meat products, which are vulnerable to economically motivated adulteration problems. To the best of our knowledge, limited studies have been performed on the quality evaluation of meat samples by using portable FTIR systems. The growing extent and complexity of the food supply chain has created a requirement for in situ determination of the meat quality and safety. Dashti et al. [50] conducted a study on the evaluation of meat authenticity by using a portable FTIR spectrometer and diffuse reflection-FTIR in combination with multivariate classification techniques. Their results showed that both portable and DR-FTIR can be used effectively for the on-site quality evaluation of meat species, such as minced beef, lamb, chicken and pork. Additionally, these results may shed light on authenticity problems related to halal meat products.

Besides the mentioned food products, nuts, honey and wine are considered valuable food products that are prone to adulteration through economic gain. In this context, various studies have evaluated the ability of portable FTIR systems to determine the authenticity and identify adulteration in these valuable food products. For instance, the detection of green pea and peanut adulteration in pistachio nuts was conducted by using portable FTIR and portable NIR systems in conjunction with SIMCA and PLSR models [51]. The results showed the strong ability of both portable FTIR and portable NIR systems to be used as alternative methodologies for the in situ and in-the-field control of high-value pistachio nuts by regulatory laboratories and stakeholders as rapid, effective and nondestructive analytical tools. In another study, Manfredi et al. [52] used portable FTIR spectrometry coupled with chemometrics for the classification of raw hazelnuts, and the authors reported that the built methodology could be successfully used for verification of the hazelnut cultivar.

A popular and important food is honey, which has been consumed for its favorable flavor and health-beneficial properties since ancient times. Honey is becoming more valuable and popular as a natural product, because of sustainability concerns and consumer demand. The quality of honey is affected by a lot of factors, such as the climate conditions, geographical conditions, floral origin, pollen type and handling and processing conditions. Honey, with its high economic importance and characteristic properties, is one the most vulnerable products for food adulteration and authenticity problems. Obviously, there is a need for high-throughput, rapid, nondestructive and low-cost methodologies to control the honey quality in the food supply chain from farm to fork. The application of FTIR spectroscopy combined with a multivariate analysis can be used as an alternative methodology to maintain the honey quality with in situ applications. In this context, Ayvaz [53] evaluated the performance of a portable FTIR spectrometer and hand-held NIR equipment with PLSR for the rapid determination of various quality parameters (ten key quality parameters) of honey, such as sucrose, glucose, fructose, reducing sugar, 5-HMF, °Brix, the moisture content, water activity, pH and free acidity). The author reported that both types of equipment performed well; however, the portable FTIR system was superior for the determination of sucrose, 5-HMF and free acidity, while the portable NIR equipment showed a better performance regarding the quantification of the Brix and moisture contents.

Besides the mentioned food products and their industrial importance, the winemaking industry is an important contributor to the economies of a lot of countries. According to previous reports, it is essential to provide thorough process control from harvest to the bottling stage to produce high-quality wines [54]. To the best of our knowledge, limited studies have been performed to evaluate the abilities of portable and hand-held FTIR systems to be used for quality control in the wine process from farm to fork. In one of these studies, Cavaglia et al. [55] employed a portable FTIR-ATR spectrometer combined with PCA and PLSR multivariate statistics to monitor the fermentation process to detect microvinifications and defective fermentations in the fermentation process. According to their results, ATR-FTIR spectroscopy combined with chemometrics has the ability to be used for the early detection of fermentation defects with at-line application possibilities. With the application of these methodologies, it is estimated that the producer may have the opportunity to maintain the product quality in a rapid, easy and accurate way by monitoring the changes in the fermentation process.

To the best of our knowledge, limited studies have been performed on the quality evaluation of cereals by using portable FTIR systems. In a thesis study, the quality of Andean indigenous grains, such as quinoa (Chenopodium quinoa), cañihua (Chenopodium pallidicaule) and kiwicha (*Amaranthus caudatus* L.), was evaluated by using both portable and benchtop FTIR spectrometers combined with SIMCA chemometrics. FTIR spectroscopy showed a strong ability to be used for the determination of adulteration, and the results were comparable with the traditional benchtop method [56]. In another contribution, Zhu et al. [57] evaluated the ability of the hand-held NIR spectrometer and portable FTIR equipment to measure the quality properties (β-glucan, starch, protein and lipids) of oats to support breeding selection. Similar predictive accuracy levels were obtained by using PLSR models with FTIR and NIR methodologies. 

**Table 1:** Overview of reported studies using Fourier Transform Infrared (FTIR) Spectroscopy to determine the authenticity, quality, safety and other essential parameters in foods.

Commodity	Type of Food Product	Parameters Measured	Data Acquisition	Type of Analysis	Equipment, Multivariate Analysis	Reference
**Fats and oils**	Edible fats and oils	Trans fat content	4000–700 cm^−1^	Quality and safety control	ATR-FTIR, PLSR	[33]
	Fats and oils	Trans fat content	4000–700 cm^−1^	Quality and safety control	ATR-FTIR, MCT detect	[34]
	Fast-foods (hamburgers, chicken tenders, French fries, apple pies)	Trans fat content	4000–700 cm^−1^	Quality and safety control	ATR-FTIR, using MCT detector	[35]
	Fats, oils and lipids extracted from fast-foods	Total trans fat	966 cm^−1^	Quality and safety control	ATR-FTIR	[36]
	Corn, cottonseed, canola, safflower, safflower, and peanut	Fatty acid composition, peroxide values, free fatty acids	4000–650 cm^−1^	Quality control	ATR-FTIR, SIMCA and PLSR	[37]
	Sacha inchi oil and vegetable oils(canola, flax, cottonseed, sunflower and olive)	Fatty acid profiles (palmitic, stearic, oleic linoleic, linolenic)	4000–650 cm^−1^	Quality control, determination of adulteration	ATR-MIR, SIMCA, PLSR	[38]
	Microencapsulated fish oil supplements	Oil and protein contents	1800–950 cm^−1^	Quality control	ATR-FTIR, PLSR	[39]
	Marine oil omega-3 dietary supplements	Major and minor fatty acid (EPA, DHA, PUFA)	650−1500 or 650−1500 and 2800−3050 cm^−1^	Quality control	ATR-FTIR, PLSR	[40]
	Edible oils and fast-food lipid extracts (hamburgers, chicken tenders/nuggets, French fries, and apple pies)	Total trans fatty acid (TFAs) contents	4000–650 cm^−1^	Quality and safety control	ATR-FTIR, PLSR	[41]
	Marine oil omega-3 dietary supplements	Major and minor fatty acids (EPA, DHA, PUFA)	4000–650 cm^−1^ and 12,500–4000 cm^−1^	Quality control	ATR-FTIR, FT-NIR, PCA, PLS-DA	[42]
	Butter and margarine	Trans fats, conjugated linoleic acid (CLA) and other fatty acids	4000–700 cm^−1^	Quality control	FTIR, FT-NIR, SIMCA	[43]
**Vegetables**	Tomato	Quality parameters (°Brix, pH, titratable acidity, fructose and glucose, and citric and glutamic acid	4000−700 cm^−1^	Quality control, maturity control	ATR-FTIR, PLSR	[44]
	Raw Potato Tubers	Sugars, asparagine, acrylamide and glutamine levels	4000–700 cm^−1^	Quality control, classification	ATR-FTIR, MIR PLSR	[45]
	Potato (Andean native)	Anthocyanin, other phenolics and sugar (glucose, fructose and sucrose	4000 and 700 cm^−1^	Quality control, classification	ATR-FTIR, PLSR	[46]
	Onion juice	Carbohydrate profiling	4000–375 cm^−1^	Quality control, classification	HCA of ATR-FTIR Spectra	[47]
	Leafy Vegetables (Chinese cabbage, water spinach, celery, and lettuce)	Nitrate content	4000–400 cm^−1^ and 1500–1200 cm^−1^	Quality and safety control	ATR-FTIR, ED-ELM model PCA	[48]
**Milk and dairy products**	Milk powder (Nonfat dry milk and skim milk powder	Melamine	4000–650 cm^−1^	Safety control, adulteration	ATR-FTIR, SIMCA	[49]
**Meats**	Minced beef, lamb, chicken and pork	Lipids, phospholipids, proteins and amino acids	3200–1000 cm^−1^	Quality control	ATR-FTIR, DR-FTIR, OCC screening approach	[50]
**Cereals**	Andean flours	Protein, amino acids	4000–700 cm^−1^	Quality control, authentication	ATR-FTIR, ATR-MIR, SIMCA	[56]
	Oat groat	β-glucan, starch, protein, and lipids	4000–650 cm^−1^	Quality control	FT-IR, FT-NIR, PLSR	[57]
**Nuts**	Pistachio nuts (Pistacia vera fruits)	Detection of green pea and peanut	4000–650 cm^−1^ and 3920–7400 cm^−1^	Quality control, determination of adulteration	FT-MIR and FT-NIR spectroscopy, SIMCA, PLSR	[51]
	Hazelnut	Lipids, proteins	3660–2500 cm^−1^ and 1850–667 cm^−1^	Quality control	FTIR, PCA-LDA and PLS-DA	[52]
	Pistachio nuts (Pistacia vera fruits)	Detection of green pea and peanut	4000- 700 cm^−1^	Quality control, adulteration	FT-IR and UV–Vis spectrometers, SIMCA, PLSR	[58]
**Others**	Honey	Quality parameters (sucrose, glucose, fructose, reducing sugar, 5-HMF, Brix, moisture content, water activity, pH and free acidity)	6250–4170 cm^−1^ (1600 and 2400 nm)	Quality control	FTIR, MIR, NIR, PLSR	[53]
	Wine (must fermentation)	Density, sugars (glucose and fructose) and acetic acid values	950–1500 cm^−1^ and 3000–3500 cm^−1^	Quality control, fermentation control	ATR-FTIR, PCA, PLSR, and PLS-DA	[55]

## 6. Conclusions

The complexity and diversity of the food supply chain make the system vulnerable to a wide variety of food adulteration and food authenticity problems. Traditional and reliable food control methodologies are generally laborious, time-consuming and require an expert as well as toxic chemicals. Evolution and progress in food control systems can be achieved through the application of state-of-the-art and rapid techniques, such as spectroscopic and nontargeted fingerprinting methodologies equipped with multivariate statistics to maintain food quality standards and control specific label requirements. This review study explained the abilities of hand-held and portable FTIR spectrometers combined with chemometrics to be used for the determination of various quality properties in food products, such as fats, oils, vegetables, milk and dairy products, meats and nuts. Previous contributions highlighted the high potential of portable systems to be used for on-site food control, since quite comparable results have been obtained with conventional techniques (confirmatory analysis), benchtop spectrometers and portable spectrometers. The application of portable and hand-held FTIR systems combined with advanced multivariate statistics can allow robust, rapid, cost-effective and in situ determination of food quality properties. As a conclusion, it is possible to state that hand-held and portable instruments have groundbreaking potential to maintain food quality and food safety in various critical segments of the food supply chain from farm to fork.

## Data Availability

Not applicable.
